# Supplementary material for: Mortality as the primary constraint to enhancing nutritional and financial gains from poultry: A multi-year longitudinal study of smallholder farmers in western Kenya
Source: PLoS One. 2020 May 29;15(5):e0233691. doi: 10.1371/journal.pone.0233691 (PMC7259595; doi:10.1371/journal.pone.0233691)
Supplement: S1 Table — (DOCX) [file pone.0233691.s001.docx]

**Supplementary table S1: Summary of quarterly household incomes (USD)**

| **Visit quarter** | **Household incomes (mean ± standard deviation)** | **95% confidence interval** |
| --- | --- | --- |
| 1 | 112.70±294.18 | 99.47-125.92 |
| 2 | 106.28±212.58 | 96.72-115.84 |
| 3 | 121.90±249.69 | 110.67-133.13 |
| 4 | 118.06±226.91 | 107.85-128.26 |
| 5 | 113.02±234.97 | 102.45-123.58 |
| 6 | 123.17±253.37 | 111.78-134.56 |
| 7 | 99.97±223.13 | 89.94-100.00 |
| 8 | 89.27±213.36 | 79.67-98.86 |
| 9 | 107.16±320.24 | 92.76-121.56 |
| 10 | 111.20±321.98 | 96.73-125.68 |
| 11 | 84.88±301.88 | 71.31-98.46 |
| 12 | 112.21±309.97 | 98.27-126.15 |
| 13 | 100.77±221.97 | 90.79-110.75 |
| 14 | 92.05±305.18 | 78.33-105.78 |
| 15 | 100.07±352.66 | 84.21-115.93 |
| 16 | 93.88±205.27 | 84.65-103.11 |
| **Cumulative Mean** | **105.41±265.46** | **93.47-117.35** |
