# Supplementary material for: Mortality as the primary constraint to enhancing nutritional and financial gains from poultry: A multi-year longitudinal study of smallholder farmers in western Kenya
Source: PLoS One. 2020 May 29;15(5):e0233691. doi: 10.1371/journal.pone.0233691 (PMC7259595; doi:10.1371/journal.pone.0233691)
Supplement: S2 Table — (DOCX) [file pone.0233691.s002.docx]

**Supplementary Table S2: Summary of quarterly income and on-farm expenses^1^**

| **Variables per quarter** | **Mean** | **95% confidence intervals** |
| --- | --- | --- |
| Household Income | 105.41 | 93.47-117.35 |
| Livestock earnings^2^ | 11.08 | 9.91-12.25 |
| Chicken sale value | 1.97 | 1.57-2.39 |
| Livestock expenses | 11.46 | 10.53-12.38 |
| Chicken expenses | 0.37 | 0.26-0.48 |
| Crop earnings | 2.04 | 1.65-2.44 |

^1^USD; ^2^Livestock excluding chicken
