# Supplementary material for: Mortality as the primary constraint to enhancing nutritional and financial gains from poultry: A multi-year longitudinal study of smallholder farmers in western Kenya
Source: PLoS One. 2020 May 29;15(5):e0233691. doi: 10.1371/journal.pone.0233691 (PMC7259595; doi:10.1371/journal.pone.0233691)
Supplement: Attachment — Submitted filename: PalmerG et al_2020.pdf [file pone.0233691.s003.pdf]

PLOS ONE

PONE-D-20-06528

Title: Mortality as the primary constraint to enhancing nutritional and financial gains from poultry: A multi-year longitudinal study of smallholder farmers in western Kenya

Summary

In this manuscript, Palmer et al are reporting the outcomes from a four-year study conducted to determine why, despite obvious reasons of keeping large chicken flocks to maximize nutritional and income benefits, the average number of chickens kept at the household level in rural western Kenya, is low. They found out that, even though there is substantial additions through hatchings, there are involuntary significant losses mainly due to mortality. Their data suggest that the smallholder households can substantially alleviate poverty through vaccination and feed supplementation as opposed to provision of more chickens.

Comments:

This is a well conducted study and the data support the conclusion. Importantly, the manuscript provides empirical data needed to address poverty reduction by managing losses due mortality to improve poultry keeping, especially by women. The manuscript is well written.

Weaknesses/Concerns: None
