# Supplementary material for: Mortality as the primary constraint to enhancing nutritional and financial gains from poultry: A multi-year longitudinal study of smallholder farmers in western Kenya
Source: PLoS One. 2020 May 29;15(5):e0233691. doi: 10.1371/journal.pone.0233691 (PMC7259595; doi:10.1371/journal.pone.0233691)
Supplement: Attachment — Submitted filename: Review comments-PONE-D-20-06528.docx [file pone.0233691.s005.docx]

**Manuscript Review: PONE-D-20-06528:** Mortality as the primary constraint to enhancing nutritional and financial gains from poultry: A multi-year longitudinal study of smallholder farmers in western Kenya

The article provides evidence of significant constrains to nutritional and financial gains from poultry production system in rural communities in many ways.

**Please see comments below**

The article is well written, and its contents are valuable and could be considered for publication in PLos One after the following comments and concerns are addressed.

1.	The title page and the manuscript body does not conform to PLos One guideline and should be formatted as such. Also, there should be page and line numbering in the entire manuscript. See guideline link: <https://journals.plos.org/plosone/s/file?id=7797/Title%20Page%20-%20ONE%20Formatting.pdf>,

2.	As stated by the authors, in spite of the large population of village chickens in rural communities in Africa, their productivity is low due to various factors including high mortality rates, especially in chicks. While village chickens do not only play an important role in the supply of protein and income to resource-poor households. It is important to recognize the relevance of rural chickens in occasions such as religious and cultural events in the African traditional settings.

3.	Other minor review comments are included in the attached manuscript for authors consideration.
