# Supplementary material for: Mortality as the primary constraint to enhancing nutritional and financial gains from poultry: A multi-year longitudinal study of smallholder farmers in western Kenya
Source: PLoS One. 2020 May 29;15(5):e0233691. doi: 10.1371/journal.pone.0233691 (PMC7259595; doi:10.1371/journal.pone.0233691)
Supplement: Attachment — Submitted filename: Response to Reviewers.docx [file pone.0233691.s006.docx]

We appreciate the reviewers’ time and effort in the critique of the manuscript and have responded to the comments below and note the specific changes in the revised manuscript.

Reviewer 1: The reviewer did not have any specific comments for change or improvement. We appreciate the comments.

Reviewer 2: The reviewer had 3 specific suggestions, each of which is addressed below and with changes to the manuscript.

2.1 *“I suggest that, instead of numbering visits (1-16), could this be reported per season, say rainy and dry season for example?”* All visits cannot be assigned to a given rainy/dry season as the actual start, duration, and intensity of the rainy seasons vary by year and many visits could not be confidently placed within a rainy/dry season. However, we identified a subset of 50 households for which visits could be placed by year into either a dry season or wet season. We then analyzed the same variables (gains/losses, source of gains/losses, sale price) by season. Based on this subset of households, there was no difference in any of the variable by season. We have added this finding to the manuscript.

2.2 *“I also would like to see some concrete discussion on how these data could be utilized to improve poultry production.”* We have modified the Discussion to emphasize the importance of preventable mortality through improved access to veterinary services. As the reviewer requests in the 3^rd^ comment, we report the minimal use of veterinary services and integrate this finding with the barriers to smallholder poultry vaccination as recently reported by Campbell et al. (references 11,24).

2.3 *“Please also comment on chicken breeds in villages and type of housing for chickens, also comment on veterinary service (available of nor available).”* Defined as “local African chicken”, these chickens are described as “ecotypes” rather than “breeds” as there is no defined strategy for maintaining genetic lines. Across ecotypes there is marked diversity, significantly greater than in defined poultry breeds. We have added this to the manuscript and provided a contemporary reference (28). We have also provided an analysis of housing: 98% of households allowed chickens to scavenge during the day; 93% maintained chickens within the human household at night with 7% in a dedicated coop or enclosure. This has been added to both the Results/Discussion. We also addressed use of any veterinary service: 12% of households reported receiving some form of treatment, vaccination, or de-worming during the survey period. To this data, we have added in the Results/Discussion reference to a second set of more complete data on a subset of 533 households in which 5.65% reported veterinary service with vaccination at 1.35%, ectoparasite control at 1.26, and de-worming at 0.42%.

Reviewer 3: The reviewer had 2 specific suggestions, each of which is addressed below and with changes to the manuscript. In addition, the reviewer recommended several minor grammatical edits to the manuscript.

3.1 “*The title page and the manuscript body does not conform to PLos One guideline and should be formatted as such. Also, there should be page and line numbering in the entire manuscript.”* We have revised the manuscript to be in compliance with PLoS One guidelines and have included page and line numbering.

3.2 “*As stated by the authors, in spite of the large population of village chickens in rural communities in Africa, their productivity is low due to various factors including high mortality rates, especially in chicks. While village chickens do not only play an important role in the supply of protein and income to resource-poor households. It is important to recognize the relevance of rural chickens in occasions such as religious and cultural events in the African traditional settings.”* We appreciate this comment as some of this significance is reflected in gifts and consumption as sources of both gain and loss of chickens at the household level. We have added this important role, with an accompanying relevant reference, to the Introduction.

3.3	*“Other minor review comments are included in the attached manuscript for authors consideration.”* We have made the suggested grammatical changes.
